# Innovative Approaches to Hepatocellular Carcinoma: Diagnostic Breakthroughs, Biomarker Integration, and Artificial Intelligence

**DOI:** 10.3390/biomedicines13102439

**Published:** 2025-10-07

**Authors:** Evangelos Koustas, Eleni-Myrto Trifylli, Panagiotis Sarantis, Michalis V. Karamouzis

**Affiliations:** 1 Oncology Department, General Hospital Evangelismos, Ipsilantou 45-47, 10676 Athens, Greece; 2 Institute of Molecular Medicine and Biomedical Research, 11527 Athens, Greece; trif.lena@gmail.com (E.-M.T.); panayotissarantis@gmail.com (P.S.); mkaramouz@med.uoa.gr (M.V.K.); 3 Academic Department of Internal Medicine, General and Oncology Hospital “Agioi Anargyroi”, National and Kapodistrian University of Athens, Timiou Stavrou 14, 14564 Kifisia, Greece

## 1. Introduction

Primary liver cancer is the fifth most common malignancy globally and the second most common cause of cancer-related deaths. This phenomenon is mainly due to the association between hepatocellular carcinoma (HCC) and viral infections of chronic hepatitis B (HBV) and hepatitis C (HCV). HCC is extremely heterogeneous, a phenomenon attributed to its multifactorial etiopathogenesis, and the annual number of liver cancer cases globally (748,300) closely resembles the number of deaths (695,900) [1]. Furthermore, there is a gender disparity regarding the incidence of HCC, with incidence and mortality rates being two to three times higher in men compared to women on a global scale, regardless of disease etiology.

Recent advancements in HCC diagnosis and treatment have notably improved patient outcomes. Long-term survival rates are 3% to 5% in most cancer registries, whereas untreated HCC has a dismal prognosis, with a <10% 5-year survival rate. The background of chronic liver disease in HCC adds significant complexity to its therapeutic management. For localized HCC, surgical resection, orthotopic liver transplantation (OLT), and ablative therapies are curative approaches at present. A better understanding of natural history, pathogenesis, and tumor biology as concerns HCC will open up new horizons in the development of novel, effective treatment options. Considering the multiple etiologies of HCC development, there is an urgent need for new knowledge regarding the molecular background of this disease that will facilitate early diagnosis, prognosis, prevention, and personalized medicine [2].

In this Special Issue, a variety of studies that focus on retail in relation to cities, including four articles and three reviews, is presented. These will be briefly described in the next paragraphs; however, it is important to clarify that it is not the purpose of this editorial to elaborate on each of the texts included in this Special Issue, but rather to encourage the reader to explore them instead. Below is an overview of the latest developments.

## 2. Diagnostic Innovations

HCC is diagnosed and evaluated using various imaging technologies. Abdominal ultrasound (US) constitutes the most common diagnostic technique, utilizing sound waves to create liver images, providing initial detection and guidance for biopsies. In recent years, a plethora of more sophisticated imaging techniques has been developed. Contrast-enhanced ultrasound (CEUS) is an innovative technique that uses microbubble contrast agents to provide real-time imaging of blood flow, identifying early arterial enhancement and subsequent washout, which is characteristic of HCC.

Non-contrast MRI has demonstrated superior sensitivity in detecting early-stage HCC compared to traditional ultrasound, reducing false positives and unnecessary procedures as a result. In addition, dynamic contrast-enhanced MRI (DCE-MRI) offers superior soft-tissue contrast resolution and can reveal detailed perfusion patterns and tissue characteristics, aiding in more accurate diagnosis. Functional MRI techniques such as diffusion-weighted imaging (DWI) can be used to assess tissue cellularity and provide insights into the tissue’s microstructure. PET/CT is not routinely utilized; however, hepatocyte-specific tracers can provide metabolic information to facilitate tumor detection and staging. CT perfusion, which is part of the evolution of traditional CT scanning, uses contrast agents to evaluate blood flow dynamics within the liver and tumors, offering insights into angiogenesis [3].

Furthermore, with the development of artificial intelligence (AI) and collective data analysis, an emerging technique called radiomics—which extracts numerous quantitative features from medical images—is extending beyond human perception to identify patterns associated with tumor aggressiveness, invasiveness, and response to treatment [4].

While advances in imaging techniques significantly augment the early detection of HCC, liquid biopsy and, consequently, the molecular profiling of tumors constitute non-invasive tools that identify tumor-specific molecular alterations, offering further opportunities in prognosis and personalized therapy. Evidence suggests that dying tumor cells release small fragments of DNA shed from cancer cells into the bloodstream, called circulating tumor DNA (ctDNA). Additionally, except for ctDNA, circulating tumor cells (CTCs) can circulate in the bloodstream. Both ctDNA and CTCs act as a “molecular fingerprint,” giving valuable real-time information without the need for repeated tissue biopsies.

A novel approach to liquid biopsy is the detection of extracellular vesicles found in bodily fluids such as blood, including exosomes and microvesicles, which carry tumor-derived molecules and can be analyzed for their contents. Long non-coding RNAs (lncRNAs) and circular RNAs (circRNAs) are non-coding RNA molecules found in extracellular vesicles, which can serve as putative biomarkers, while the implementation of AI could identify new druggable targets that cannot be identified via traditional linear statistical analysis. Moreover, epigenetic markers such as DNA methylation can be detected in cfDNA and can act as emerging biomarkers for patients with HCC. These provide real-time information about the molecular characteristics of a tumor, enabling early detection, the monitoring of disease progression, the assessment of minimal residual disease, and the evaluation of treatment response without the need for repeated tissue biopsies [5].

Collectively, these novel liquid biopsy approaches form minimally invasive strategies that enable comprehensive molecular characterization of hepatocellular carcinoma. By providing real-time data on genetics, heterogeneity, and cancer cell dynamics, they hold potential for the early detection of HCC, prognostic assessment, therapeutic monitoring, and personalized treatment.

## 3. Multi-Omics and AI Integration

Machine learning models integrating multi-omics data have achieved an area under the receiver operating characteristic curve (AUC) of up to 0.85, aiding in early diagnosis and personalized treatment strategies. Multi-omics data provide a detailed, multi-layered view of HCC, while AI algorithms can identify patterns and relationships within these diverse datasets to uncover the complex molecular underpinnings of the disease. This allows for the identification of distinct HCC subtypes with unique molecular signatures.

Analyzing multi-omics data with AI helps in understanding patient-specific disease mechanisms and predicting responses to different therapies, such as immune checkpoint blockade. AI algorithms can integrate multi-omics data to identify existing drugs that target specific HCC-associated pathways or biomarkers [6]. The integration of multi-omics with AI facilitates the discovery of key biomarkers and driver mutations, which are crucial for enhancing risk prediction models and guiding targeted therapeutic interventions.

## 4. Treatment Breakthroughs

Recent breakthrough treatments for HCC include the successful combination of the TACE procedure with systemic therapies using checkpoint inhibitors and targeted therapy, reflecting progress in both systemic and locoregional therapeutic strategies [7]. These developments have not only improved clinical outcomes such as overall survival and progression-free survival for patients with advanced disease but have also expanded the range of therapeutic options available for earlier stages of HCC.

The efficacy of combined double checkpoint inhibitors, including anti-PD-L1 and anti-CTLA-4 (nivolumab and ipilimumab, respectively), was evaluated in CHECKMATE-9DW. This study demonstrated improved OS in advanced, unresectable, or metastatic HCC, outperforming standard therapies such as sorafenib and lenvatinib [8]. The IMbrave050 trial revealed that adjuvant therapy with atezolizumab and bevacizumab post-surgery significantly reduced recurrence risk in high-risk HCC patients [9].

In addition, investigational agents such as SCT-I10A, an anti-PD-1 antibody, combined with SCT510, have demonstrated promising efficacy in advanced HCC, offering potential alternatives to current treatments [10]. Recent advances in cell-based and nanotechnology approaches, such as GPC3-targeted CAR-T cell therapies and nanotechnology-based drug delivery systems, are currently under study, with the aim of these being to enhance treatment precision and minimize side effects [11].

## 5. Key Knowledge Gaps in HCC Diagnosis and Treatment

The major challenge for HCC remains the early detection of the disease. Even with advanced imaging and new biomarkers, early-stage HCC detection often remains difficult in routine clinical practice, especially in high-risk populations with hepatic cirrhosis. The sensitivity and specificity of liquid biopsies and molecular biomarkers still require validation in large, diverse cohorts.

In addition, heterogeneity and molecular subtype classification appear to act as resistance mechanisms against various therapies such as immunotherapy. It is a widely acknowledged fact that HCC is highly heterogeneous at the genetic and molecular levels, but a comprehensive molecular classification system that can guide personalized therapeutic approaches is still lacking, as the association between specific molecular alterations and the treatment response remains unclear.

Moreover, the mechanisms driving immunotherapy resistance and how to overcome them are not fully understood. Optimal therapeutic approaches are required, which could include combinations of different therapeutic modalities. More clinical trials are needed to establish guidelines tailored to patient-specific factors in collaboration with prognostic and treatment-selection biomarkers.

In order to accurately identify the therapeutic plan that is most beneficial for patients, it is necessary to use current markers; however, markers such as AFP have limited predictive value. The recurrence and progression of advanced disease remain high even after surgery or ablation, and effective strategies to prevent or treat recurrent HCC are limited. The integration of AI and multi-omics data shows promise; however, their real-world clinical utility, reproducibility, and integration into workflows are still in their early stages. Large-scale validation and standardization of these techniques are therefore required.

## 6. Conclusions

Advancements in AI have facilitated personalized treatment plans by analyzing vast amounts of clinical, imaging, and genomic data. Moreover, multidisciplinary teams are essential in managing HCC, ensuring comprehensive care through collaboration among specialists. These advancements underscore a shift towards more effective, personalized, and less invasive therapeutic approaches in HCC management, offering advanced tools to improve patient outcomes. Advanced and novel imaging techniques, combined with computational methods, AI, and new biomarker integration, are expected to significantly enhance the accuracy and early diagnosis of HCC.
